# A sense of connectedness, transcendent experiences, and insights for compassionate action emerge through an international collective labyrinth walk with a shared intention during the COVID-19 pandemic

**DOI:** 10.3389/fpsyg.2023.1232784

**Published:** 2023-11-14

**Authors:** Jocelyn Shealy McGee, Christine Katzenmeyer, Stephanie Clintonia Boddie, Rebecca Meraz, Sinai Wood

**Affiliations:** ^1^Diana R. Garland School of Social Work, Baylor University, Waco, TX, United States; ^2^The Legacy Labyrinth Project, Denver, CO, United States; ^3^George W. Truett Theological Seminary, Baylor University, Waco, TX, United States; ^4^School of Education, Baylor University, Waco, TX, United States; ^5^College of Human Sciences, Institute for Gender Studies, University of South Africa, Pretoria, South Africa; ^6^Louise Herrington School of Nursing, Baylor University, Dallas, TX, United States; ^7^University Libraries, Baylor University, Waco, TX, United States

**Keywords:** labyrinth walking, contemplative practices, flourishing, wellbeing, connectedness, transcendence, spirituality, consciousness

## Abstract

**Introduction:**

Labyrinth walking is an integrative contemplative practice that aims to engage the body, heart, mind, and spirit. In this article, qualitative findings from the first year of a mixed methods study on collective labyrinth walking with a shared intention are described. This form of labyrinth walking is distinct in that it is a social contemplative practice. It expands upon most of the labyrinth walking research to date which has been focused upon the individual. More specifically, practitioners walk labyrinths together in solidarity with the same intention in mind during collective labyrinth walking. This practice can be used locally (i.e. practitioners walk the same labyrinth together for the same reason) or non-locally (i.e. practitioners walk different labyrinths for the same reason together in different locations). The study is unique in that it took place at the height of the COVID-19 pandemic which was a time in recent history that evoked fear, uncertainty, grief, isolation, and disconnectedness for many persons around the world.

**Methods:**

This sample in this study was comprised of 461 participants from 19 countries who collectively walked labyrinths together with a shared intention on World Labyrinth Day 2021. Most participants were women in middle to later life from the United States. Data was collected through an anonymous online survey and analyzed using the qualitative methodology of interpretive phenomenological analysis.

**Results:**

Three predominant themes emerged from practitioners’ narrative accounts of their lived experiences: (1) multiple forms of connectedness (i.e., intrapersonal, interpersonal, transpersonal, labyrinth connection) were cultivated through collective labyrinth walking with a shared intention; (2) practitioners reported qualities associated with “transcendent” experiences during this experience (i.e., boundlessness, ultimacy, transcendence, connectedness, positive emotions); and (3) practitioners had insights for compassionate action.

**Discussion:**

Findings suggest that collective labyrinth walking with a shared intention can contribute to individual and group flourishing during times of distress. Quasi-experimental and experimental research designs are needed to build on this exploratory developmental research and are described in this article.

“There is so much fear being perpetuated during these COVID times. So, the question of how to make this change and let go was revealed as I stepped out of my mind and into my heart. I felt others doing the same collectively. Compassion for others, self-compassion and some humor from the Universe presented itself as I walked the labyrinth.”

–Participant

## Introduction

Qualitative findings from the first year of a 3-year long mixed methods study on collective labyrinth walking with a shared intention are described in this article. This form of labyrinth walking is distinct in that it involves a shared social experience, either locally or non-locally, in which practitioners walk at the same time in solidarity for the same reason. The study is significant in that it is the first to examine the experience of collective labyrinth walking with a shared intention as compared to prior research on labyrinth walking which has been predominantly focused upon individual labyrinth walking. The study is unique in that it took place at the height of the COVID-19 pandemic which was a period that evoked fear, uncertainty, grief, and isolation for many persons around the world. The study is innovative in its community-engaged field-based design which was aimed at better understanding a real-world scenario in which a contemplative practice was implemented as compared to a lab-based study. The purpose of the study was to learn about labyrinth walking practitioners’ thoughts, feelings and insights while collectively walking labyrinths, in multiple locations, at the same time, a little over a year after the COVID-19 pandemic began on World Labyrinth Day 2021. The impetus for the study was the labyrinth community’s dedication to walking labyrinths together as an expression of solidarity during an international crisis as well as dedication to research on learning more about this contemplative practice. By way of background, the psychosocial impacts of the first year of the COVID-19 pandemic are discussed to provide context for the study followed by an introduction to the field of contemplative science and practice. A summary of prior research on labyrinth walking is provided as well as a description of the distinct practice of collective labyrinth walking with a shared intention.

## Background

### Psychosocial impacts of the COVID-19

The first year of the COVID-19 pandemic was a challenging time for many in the recent history of the world. The prevalence of disease and death on a global scale was unprecedented as the virus spread. In addition, a major toll was taken on the financial, social, and emotional health of many (Brooks et al., 2020). Rates of depression and anxiety in the USA tripled as compared to pre-pandemic levels (Czeisler et al., 2020). Similarly, international researchers reported high levels of anxiety, depression, sleep disturbance, stress, and emotional trauma in the community leading to a call for increased mental health resources as a public health response (Bao et al., 2020; Jia et al., 2020; Li et al., 2020; Rajkumar, 2020; Giel et al., 2021; Lee Y. et al., 2021; Panda et al., 2021; Taquet et al., 2021; Matsumoto et al., 2022).

Individuals, families, and communities sought ways to protect their health and to maintain their wellness during the COVID-19 pandemic in the context of distress and social isolation. Wellness, according to the World Health Organization (2023), is a “state of physical, mental and social wellbeing and not merely the absence of disease or infirmity.” It is a “conscious, self-directed, and evolving process of achieving full potential” (National Wellness Institute, 2023). As such, some persons engaged in various forms of contemplative practice for comfort and guidance as they learned to navigate the unique challenges of the pandemic (Achepohl et al., 2022; Lekhak et al., 2022). Labyrinth walking practitioners turned to this unique contemplative practice as a means of coping, maintaining wellness, and cultivating flourishing.

### Contemplative science

A goal of the interdisciplinary field of contemplative science, according to Dorjee (2016), is to gain a better understanding of “the core capacities, processes and states of mind modified by contemplative practice” (p. 9). As such, this interdisciplinary field seeks to understand the capacity of the human mind for self-regulation through metacognitive processes which can be influenced by a person’s existential awareness, motivations, and intentions—as well as the contextual factors associated with the practice they are engaging with (Dorjee, 2016).

Contemplative scientists are interested in learning how various contemplative practices serve to cultivate a range of desirable outcomes. For example, some practices are aimed at increasing a person’s capacity for loving kindness and compassion (e.g., Hofmann et al., 2011), joy and equanimity (Wallace, 1999), and acceptance and non-reactivity (Bishop et al., 2004; Baer et al., 2008). Other practices seek to generate a sense of meaning and purpose and to cultivate specific strengths and virtues (Dreyfus, 2002; Dahlsgaard et al., 2005; Dorjee, 2016).

Contemplative scientists are also interested in studying the shifts and modes of existential awareness that may be induced by a contemplative practice. A practitioner’s level of awareness, when first learning how to engage in a specific practice, may be more consistent with their typical or habitual thought patterns. With practice, however, an increased openness to insights into the nature of the self and reality may occur (Gampopa, 1998; Dorjee, 2016). Best practices for contemplative science research utilize first- and second-person methodologies as well as third person measurements of behavioral and physiological data (Varela et al., 1991; Varela, 1996; Davidson and Kaszniak, 2015; Lutz et al., 2015).

### Contemplative practice

The term contemplative practice encompasses a broad-array of mind-body integrative practices each having unique roots within cultural-spiritual and historical contexts. An overarching goal of contemplative practices is to enhance wellbeing through cultivating the recognition of the connections people have to each other (Komjathy, 2015). Inherent in this goal is the desire for cultivating compassionate responses toward the self, others, and the world.

Perhaps the most notable development within the broader field of contemplative practice is the growth and interest in mindfulness meditation. This area of research has been highly influenced by Kabat-Zinn (2003) among other researchers (Lee J. et al., 2021). Mindfulness meditation has shown promising results in a myriad of arenas, including clinical, social, and industrial-organizational psychology as well as education, social work, healthcare, and related disciplines. Centering prayer, Lectio Divina, and labyrinth walking are a few other forms of contemplative practices, although there is less research on these practices as compared to mindfulness meditation.

One classification system for the variety of forms of contemplative practice is depicted in the Tree of Contemplative Practice (Center for Contemplative Mind in Society, 2021a,b). In this model, the roots of the tree symbolize two foundational purposes of contemplative practice: (1) increasing personal awareness; and (2) cultivating a sense of connection with the divine (e.g., God, a higher power, the universe, the earth, etc.). The branches on the Tree of Contemplative Practice are a way to categorize the forms of contemplative practice. For example, there are branches that depict practices involving movement (e.g., labyrinth walking, dancing, walking meditation, etc.), stillness (e.g., meditation, centering, etc.), and creativity branches (e.g., singing, journaling, improvisation, etc.). There is notably overlap between the forms and desired outcomes of contemplative practices. The focus of the current study, collective labyrinth walking with a shared intention, could be placed on the ritual/cyclical, generative, movement, meditative, and even activist (e.g., social justice related) branches.

### Labyrinth walking

Labyrinths beckon practitioners to step onto a meandering, often unicursal path, patterned after sacred geometry, to embark on a metaphorical spiritual journey. Historians discovered that labyrinths have been used in many cultures for the last 2,000 years (although there is some evidence of the labyrinth symbol being used as long as 4,000 years ago), beginning in Crete and spreading to most continents. According to historians, such as Jeff Saward, the earliest reliably dated labyrinths have been found in Southern Europe. These labyrinths were typical of a classical style with a series of seven concentric pathways surrounding a central goal.

In the last 30 years, the ancient practice of labyrinth walking has been revived through the work of Artress (1995, 2006, 2020) among others. Since then, this practice has grown in popularity as an adjunctive process to various types of psychotherapy and as a spiritual practice (Hong and Jacinto, 2012). There is evidence that individual labyrinth walking serves to quiet/focus the mind, foster peace, reduce stress, and improve physical, emotional, and spiritual health (Yang, 2003; Zucker, 2016; Behman et al., 2018; Weaver et al., 2018; Butcher, 2023). Little is known, however, about how these benefits translate to community or social outcomes which is one of the significant aspects of the current study.

Modern labyrinths are found in religious and secular settings such as churches of all faiths, universities, hospitals, prisons, community centers, and backyards, among other locations (Abdallah-Baran, 2003; Peel, 2004; Weigal et al., 2007; Gersbach, 2008; Hong and Jacinto, 2012; Johnson, 2013; Heard et al., 2015; Zucker et al., 2016; Lizier et al., 2018). Although modern labyrinths come in many forms and designs, one of the most familiar is the 11-circuit Chartres style labyrinth, named for the medieval pavement labyrinth found in the nave of the Chartres Cathedral in France. Another well-known modern labyrinth was constructed in the nave of Grace Cathedral in the United States and is a replica of the Labyrinth at Chartres. A third notable labyrinth is a “green rooftop labyrinth” at the American Psychological Association (APA) headquarters in Washington, DC, USA.^1^ This labyrinth was designed to provide a place for employees and visitors to reflect and as a means for reducing pollution in local waterways.

Other forms and designs of modern labyrinths include physical labyrinths in the ground, virtual labyrinths, finger labyrinths, paper labyrinths, and more. There are no financial costs for walking a labyrinth which make it a potentially scalable contemplative practice. Labyrinth walking reaches the far corners of the technological spectrum from low-tech to high-tech. An example of a low-tech labyrinth is when a person draws a labyrinth design in the dirt (or sand) with a stick, then walks it. Another example of a low-tech labyrinth is a paper handout with a labyrinth printed on it whereby a person can “walk” it by using their finger to trace the pattern. An example of a high-tech labyrinth would be the vision of Sandra Wasko-Flood in Angel Fire, New Mexico. Her prototype for a PEACE labyrinth is projected onto the floor by liquid crystal display (LCD) and can be modified based on desired labyrinth design, color, size, and music. Examples of other options for labyrinth walking are using a finger type labyrinth on an app for a smartphone, carved finger labyrinths in wood, and labyrinths illuminated by solar power for walking at night. There are also labyrinths made from recyclable materials (i.e., crushed computer discs (CDs), recycled bottle glass, old pottery, used shoes, tires) and natural materials (i.e., dried flowers from bouquets no longer being used) which allows for the production of eco-friendly labyrinths. The Labyrinth Society^2^ has curated a useful downloadable resource on research and presentations on this contemplative practice.

### Collective labyrinth walking with a shared intention

Most of the research on labyrinth walking to date, has examined this practice from an individual or more Western perspective. In the current study, we have focused on collective labyrinth walking with a shared intention which involves 3 or more people walking a labyrinth or labyrinths in solidarity for an agreed upon purpose or reason. This form of labyrinth walking can occur locally (i.e., all walkers are in the same place) or non-locally (i.e., walkers are located in different places even though they are walking at the same time with a shared purpose or intention). Shared intentions must be of a prosocial nature and about improving circumstances for an identified people group that the collective is concerned about.

A short meditation is usually conducted before beginning a collective labyrinth walk. This meditation is aimed at unifying the hearts and minds of the practitioners who are walking. Practitioners are instructed to state the shared intention silently or out loud before they step onto a labyrinth to walk the path toward the center or “heart,” also called the rosette. Upon arriving at the center of the labyrinth, practitioners are encouraged to repeat the shared intention again verbally or silently to themselves. In the center, practitioners are asked to begin to feel the emotion of the intention as if it was already completed and to release it. The practitioner remains in the center as long as they want to and walks the path out of the labyrinth when ready. At this point, the practitioner is advised to be present to what is being received in their mind or heart and receptive to any ideas, feelings, and/or sensations that emerge without analyzing or judging.

After the walk, it is recommended that a journal be used to write about what happened during the walk. The practitioner should be encouraged to recall their experience, noting any changes in their thoughts, feelings, and sensations. Possible reflections include how they felt after “releasing” the intention in the center of the labyrinth, as compared to before releasing it, and new insights or ideas they had stemming from their collective labyrinth walking experience. Individual journaling is for personal reference and for sharing when a collective comes together to discuss what came up for each member during the walk whether in-person or online.

## Materials and methods

The project was reviewed by the Institutional Review Board (IRB) of Baylor University (Reference Number 1711129). The data was collected through an anonymous online questionnaire. No personally identifiable Research Health Information or Personal Health Information was collected.

### Research design and sampling strategy

A convenience sampling strategy was employed in this study and data collection occurred on World Labyrinth Day (WLD) which was held on 1 May 2021. Inclusion criteria were: (1) participated in a labyrinth walk on World Labyrinth Day 2021; (2) signed up for the research study before WLD to receive instructions; (3) age 18 or older; (4) English reader; and (5) willingness to complete an anonymous questionnaire online. Exclusion criteria were no internet or cellular access to complete the questionnaire associated with the study.

### Procedure

#### Recruitment

The Legacy Labyrinth Project (LLP), a community organization dedicated to the practice of labyrinth walking and sponsor of the study, was responsible for recruitment and worked in close collaboration with the researchers on this study. Information about the study was distributed through social media outlets, email, study flyers, website announcements, word of mouth, and presentations. Other well-known labyrinth organizations supported recruitment efforts such as The Labyrinth Society (2023), Veriditas^3^, and The Australian Labyrinth Network.^4^ The organizers of World Labyrinth Day were supportive of the research study and provided outlets for recruitment.

#### Instructional resources

The Legacy Labyrinth Project (LLP) developed two instructional videos for the study which were emailed to participants the day before WLD 2021. The goal of the first video was to teach participants a brief meditation to becoming more calm and more focused before participating in collective labyrinth walking. The goal of the second video was to teach participants how to walk a labyrinth with a shared intention based on the work of McTaggart (2007). Participants also received a video developed by The Labyrinth Society on how to walk a labyrinth safely during the COVID-19 pandemic (based on what was known about transmission at the time).

#### Instructions for participating in the study

There were three steps for practitioners to complete in sequential order in this study. First, they were asked to do the brief meditation. Second, they were asked to reflect on the intention below that was developed by a committee of expert practitioners who served in advisory capacity for the study.

*In this year of suffering and uncertainty around the world, my intention is to walk a labyrinth with others on World Labyrinth Day to receive insights that can influence change.*

Finally, participants were asked to walk a labyrinth of any kind, wherever they were in the world, before completing an online questionnaire within 48 h of doing their labyrinth walk. There were no restrictions on the location of a practitioner’s labyrinth walk or the form of labyrinth they utilized (i.e., finger labyrinth, labyrinth on the ground, canvas labyrinth, etc.). However, practitioners were asked to walk labyrinths on the same day around 1:00 PM in their respective countries and regions with the shared intention noted above.

#### Questionnaire

A link to complete an anonymous questionnaire was sent out to practitioners to complete after their collective labyrinth walk. The questionnaire took approximately 15–30 min to complete. There were no financial or in-kind incentives for participating in the study. No electronic data on location of participants or other electronic identification was tracked.

### Data collection

An online data collection tool was utilized to collect qualitative and quantitative data (Qualtrics©, Provo, UT, USA). The measures for this study were: (1) a short investigator-developed demographic questionnaire which included questions about experience walking labyrinths as well as other information about each practitioner’s labyrinth walk; (2) three open-ended narrative questions about practitioners’ experience collectively walking a labyrinth with a shared intention; and (3) three standardized self-report measures. For the current article, we report only qualitative findings from the three open-ended questions.

Practitioners were invited to complete the questionnaire online within 48 h of collectively walking a labyrinth with a shared intention on World Labyrinth Day 2021. The questionnaire could be taken on a computer or on a smartphone. Responses went directly to the academic research team for analysis. The data was housed on a password protected server.

### Measures

Qualitative data was obtained through three open-ended questions. Participants were asked: (1) to share any insights they received during their collective labyrinth walk with a shared intention, if any; (2) what they envisioned as a plan for following up on their insights, if at all; and (3) their general experience participating in a collective labyrinth walk with a shared intention.

### Data analysis

To gain a deeper understanding of the themes emerging from the narratives of practitioners, interpretative phenomenological analysis (Alase, 2017) was utilized. This qualitative research methodology is advantageous for fostering examination of a person’s inner-most deliberations while making interpretations of the meaning of their lived experiences (Alase, 2017; Smith et al., 2022). It is particularly well-suited for learning about complex emotional and spiritual experiences (Pietkiewicz and Smith, 2014).

The narrative data from this study were uploaded to NVivo (QSR International Pty Ltd, 2022) for analysis. Three members of the research team gained insight into the data by individually reading participants’ narratives multiple times, making notes, and then developing initial codes that represented common words or sentences derived from the data. The team discussed the merits of each code and whether to retain, refine, merge, or eliminate codes over four team meetings. The agreed upon codes were grouped into “meaning units” that captured the essence of participants’ lived experiences. These codes and their definitions served as the basis for forming themes. To reduce bias, two members of the team, without personal experience with labyrinth walking, independently conducted line-by-line coding of the narratives for analysis. A third team member, who had experience walking labyrinths, served as an arbiter in case there was disagreement between the two primary coders.

The team conducted four additional meetings to process insights from individual analyses and to triangulate the data (Alase, 2017; Nowell et al., 2017). Next, the team interpreted the data around the themes which gave meaning to what practitioners shared about this collective labyrinth walk. This iterative process of text-to-code and code-to-code revision was conducted to increase the trustworthiness of the findings (Merriam, 2002; Creswell, 2013). Finally, a committee of expert labyrinth walking practitioners provided input on the interpretation of the themes.

### Demographic characteristics of participants

A total of 461 practitioners completed the online questionnaire. Although most were from the United States, practitioners from 19 countries were represented. Most practitioners were experienced labyrinth walkers with 96.3% having previously walked a labyrinth. The average age was 63 (SD = 10.726). Most practitioners self-identified as “Caucasian or White” (86.6%) although a small percentage considered themselves to be a “person of color” (e.g., “Black,” “Latinx or Hispanic,” “Indigenous,” “Asian,” “mixed racial identity”) or did not identify with any of these categories. Most practitioners self-identified as “female” (87.3%), with fewer who identified as “male” (11.9%), and the remainder preferring not to answer this question. There were people from all the world religions, persons who considered themselves spiritual and not religious, and persons who considered themselves as agnostic.

## Findings

Three predominant themes emerged from practitioners’ narrative accounts of collectively walking labyrinths with a shared intention: (1) a sense of connectedness (intrapersonal, interpersonal, transpersonal, and connectedness with the labyrinth they were walking); (2) qualities associated with a “transcendent” experience; and (3) insights into ways to cultivate social and communal flourishing. The themes are presented below in order of prevalence in this sample of practitioners.

### Theme 1: A sense of connectedness

Practitioners reported experiencing multiple forms of connectedness. Subthemes for connectedness were: (1) intrapersonal connectedness (i.e., within oneself); (2) interpersonal connectedness (i.e., with others); (3) transpersonal connectedness (i.e., with the divine, God, the Earth, nature); and (4) connectedness with the labyrinth they were walking.

#### Intrapersonal connectedness

For some practitioners, collective labyrinth walking with a shared intention led to a sense of feeling more integrated as a person. For example, a practitioner shared, “This experience brought (me) to the (realization) that I had to put my wellbeing first in my life.” Another example from a practitioner was, “I felt my heart open more and had a sense that my soul had purpose. My best days are in front of me. The Universe has my back.” This theme was important given that most practitioners in the study had reported feeling distressed and disconnected from themselves because of the COVID-19 pandemic and quarantine initiatives.

#### Interpersonal connectedness

A heightened sense of interpersonal connectedness was shared by practitioners during their labyrinth walk. The forms of interpersonal connectedness reported in this study were to family, friends, deceased loved ones and ancestors as well as fellow labyrinth walkers who were participating in this experience, practitioners’ local communities, and people around the world who were experiencing suffering as a consequence of the pandemic.

### Friends and family

Interconnectedness to friends was demonstrated in the following quote by a practitioner, “I felt a greater connection with the many friends and other people to whom I sent love and good wishes to even though we were not together (physically).” Another example was, “As I rounded the final bend (of the labyrinth) and I noticed other walkers already in the center of the labyrinth, I was overcome with a rush of emotion. I felt joyful and grateful. Somehow, I KNEW that what I was experiencing was shared by others.” Still another shared, “I was filled with joy and gratitude at being reunited on the labyrinth with four dear friends. It energized me to think that we, along with hundreds around the globe, were united in a common experience, many of us holding the same intention.” This level of perceived connection with others was notable given that many practitioners reported feeling physically and socially isolated and disconnected from family, friends, and other persons prior to their labyrinth walk.

### Deceased loved ones and ancestors

Connectedness with deceased loved ones and ancestors were often reported by practitioners. For example, a practitioner expressed, “I had a sense of anticipation and deep connection with my deceased parents.” Another practitioner shared, “Yesterday was my late Father’s 105th birthday. My heart was deeply moved when I became aware of his presence.” Another expressed having a sense of connection with deceased members of their church, “As the pastor here…I don’t know many of those buried in our memorial garden, but I certainly know their heirs. It was moving to connect with those folks (deceased parishioners) while walking and recall the stories that I’ve heard and offered prayers for their eternal rest and peace for their descendants.”

### The community, other labyrinth walkers, and the world

For some practitioners, a sense of connectedness to their communities and the world was experienced. For example, a practitioner shared, “The wisdom I received is how to deal graciously and constructively with the tension among people in the community where I live about the safety measures that are required for the pandemic.” Another practitioner shared, “I immediately felt attuned with all of the other labyrinth walkers today even though we were not in the same place.” Still another participant wrote, “I felt connected to everyone (around the world) during these challenging times.”

#### Transpersonal connectedness

In this study, we defined transpersonal connectedness as feeling connected to something larger than oneself. Transpersonal connectedness most often had to do with feeling connected to the divine or a higher power (e.g., God, gods, spirit, universe, etc.) or something metaphysical (e.g., energy, consciousness, etc.). Close to two-thirds of the practitioners’ narrative responses suggested that they experienced a transpersonal connection. For instance, a practitioner shared, “I felt peace, connected to God and encouraged that people all over the world were walking the labyrinth as one.” Another shared, “The connection with Spirit is strong, even in these seemingly difficult times.” Another shared, “I felt such a connection…to the ‘fierce and tender’ love of the Divine moving through me… (during the collective labyrinth walk).”

Another form of transpersonal connectedness reported by practitioners was to the Earth or Nature. For example, a practitioner shared, “Each inward breath (while walking the labyrinth) brought me a sense of peacefulness that came directly from the Earth itself.” Another practitioner shared, “I was filled with gratitude and connection to the Earth and others walking that day.” Another wrote: “I felt a deep love and forgiveness for all of Creation.” Nature or earth-based connections were experienced viscerally by some participants. For example, a practitioner explained: “My feet and legs were vibrating with Earth energy…it was incredible…the connectedness.” Another participant shared: “I liked very much the connection with the Earth. I could feel my own roots going down in the earth as I walked this geometric figure (labyrinth).”

#### Connection with the labyrinth

A sense of being connected to the labyrinth was reported by most practitioners. A poignant example of a labyrinth connection was shared by a participant, “I experienced a deep connection to the Now and the incredible power of being within (hugged by) the center (of the labyrinth) where I felt others across the world standing.” Another participant shared, “I had a dream last night…that connected to the last petal (of the labyrinth) today. That last petal is often very quiet for me, even today but it was also very strong.”

Labyrinth connections were imbued with meaning and healing for some as expressed in this quote, “The experience today, connecting with a complete stranger on a mainly empty shoreline, reaffirmed my love and commitment and connection with the labyrinth as a power and place and source of healing.” Another shared,

*No matter the type of labyrinth or the form of the labyrinth, they all connect to the energy of the labyrinth. I really sensed this today.*

### Theme 2: Collective labyrinth walking as a transcendent experience

There are several qualities that may present when a person believes they have had a transcendent experience. These qualities are a sense of transcendence, ultimacy, boundlessness, connectedness (previously discussed), and positive emotions (Pargament et al., 2014; Magyar-Russell et al., 2022). Any of these qualities can emerge singularly or simultaneously during a transcendent experience. Below we describe practitioners’ experiences during collective labyrinth walking with a shared intention.

#### Transcendence

Transcendence, may be described as the perception of having been a part of something bigger than the self that goes beyond one’s ordinary day-to-day existence. A sense of transcendence may occur when a person believes they have encountered a deity or deities (i.e., God, gods, etc.), although transcendence occurs outside of religious connotations. An example of transcendence from a practitioner’s narrative was, “I felt very expanded and truly connected to something bigger than myself.” Another was, “My experience was a transmutation of energy that sent ripples out from me…into the cosmos…it was much bigger than me.”

#### Ultimacy

Ultimacy may be described as the perception that an experience was “really, real” or revelatory of a deeper truth (Geertz, 1966). An example from a practitioner narrative was, “I felt the sky, a glorious azure…I was aware of all the labyrinth walkers around the world. I felt the connection, even though we were walking in different time zones…I felt privileged to be able to participate. I felt fully alive and present (in this moment).”

#### Boundlessness

Boundlessness may be described as involving the perception of moving beyond the limits of ordinary time and space. It is a perceptual shift away from Chronos time (ordinary time and space) to Cairo’s time (a more expansive experience of time and space). An example of boundlessness from a practitioner narrative was, “I lost track of time and space and had a deep sense of calm walking (the labyrinth).” Another practitioner shared, “Upon entering the labyrinth, it felt like time wasn’t important…I had stepped out of the normal confines of time and space.” A third example from a practitioner’s narrative was, “This (experience) felt like an open day…there were no barriers, if only for a moment, between that which was, that which is, and that which will be.”

#### Positive emotions

A person may experience a range of positive emotions during a contemplative practice. For example, emotions such as uplift, awe, humility, mystery, gratitude, joy, peace, and compassion, among others, may be classified in this way. In this sample, the most frequently endorsed emotion was gratitude, “I found myself a bit weepy with a deep sense of gratitude (during collective labyrinth walking).” The emotion of uplift is reflected in this practitioner’s quote, “…I felt lifted up out of my body with dignity and determination and out of the sadness I had been experiencing because of COVID.” Peace was another frequently endorsed emotion as shared by a practitioner, “…I had an overpowering sense of peace envelope me.” Joy and a sense of release was experienced by a practitioner, “I stopped and looked down to see the labyrinth shimmering in dappled light…I could almost feel the heat emanating from the surface. It only lasted a few minutes, but oh…the joy and release and relief I experienced.” Multiple positive emotions were experienced simultaneously during collective labyrinth walking with a shared intention as reported by a practitioner, “I felt a deep sense of gratitude, love, and peace from myself for others.”

### Theme 3: Compassion for action

Compassion for action was the third theme emerging from practitioners’ narratives of their experience in this study. This theme involved an increased level of awareness of the suffering other people might be experiencing as a consequence of the COVID-19 pandemic, a sense of compassion, and a desire for action. Increased awareness of the potential suffering of others is demonstrated in this quote from a practitioner, “I gained insights about addressing the HUGE suffering so many are experiencing.” Bettering oneself was regarded as an essential component of compassion for action by some practitioners as expressed in this quote, “I will continue with my efforts to bring healing to myself and others through meditation and mindfulness and labyrinth walking with compassion.” Another practitioner echoed a similar sentiment, “Healing the earth needs to start with me.”

Several practitioners reported that they were planning in engaging in direct action after receiving insights from collective labyrinth walking. An example of this sentiment follows, “I work with a group called (deleted for confidentiality). We meet buses coming in from (deleted for confidentiality) to offer a small packet of food and water to asylum seekers. We feel it is important to welcome these seekers with small practical offerings. Today’s walk encouraged me to continue with this.” Another practitioner shared a desire to translate their insights from their collective labyrinth walking experience into the actions needed for planting a community garden to address food insecurity. This practitioner went on to explain, “I am involved in helping finish this labyrinth garden and caring for it, in a community project… I’m hoping to coordinate an event to showcase the gardens on the South side of my town which was historically red-lined.” Another practitioner shared, “I am very concerned about the children all over the World who are being deprived of a joyous childhood and receive limited education.” This practitioner goes on to say, “I plan to do something about this (social issue) now that I am aware of my heart.”

Collective labyrinth walking with a shared intention was viewed as a form of non-traditional or spiritual activism by some practitioners as reflected in the following quote, “I will continue to speak out against injustice, will protest, and walk labyrinths for the good of others.” Another practitioner remarked, “As I prepare to retire…I will continue to do my justice work and will be able to hold sacred space (through labyrinth walking) to encourage other people who are doing the frontline work.” The idea of collective labyrinth walking with a shared intention being a form of prayer was often reported as demonstrated in a practitioner quote, “I will include water in my prayers while I walk labyrinths and share its importance with others.”

The subtheme of strengthening relationships among diverse people was expressed by practitioners. For example, a practitioner shared that they had received the insight that they needed to be “working toward creating peace among the people of the World.” Another practitioner expressed, “I plan to include greater inclusivity in thought and engage in conversation with a more diverse group of people…I am concerned about what happens when societies get too comfortable and complacent…when change only incorporates one point of view.” Another shared, “There is a need for the ongoing engagement and dialog around the impact of Colonization on First Nation people and movement for reparations. I recognized this during my labyrinth walk today.”

## Discussion and implications

The current study explored individual responses to the social contemplative practice of collective labyrinth walking with a shared intention within the context of the COVID-19 pandemic. Findings suggested that the practice of collective labyrinth walking with a shared intention can lead to a sense of connectedness through a transcendent or sacred experience that may spur insights and ideas for addressing human suffering with compassionate action.

Practitioners, in the study, reported experiencing various forms of connectedness during their collective labyrinth walking experience (i.e., intrapersonal, interpersonal, transpersonal, connectedness with the labyrinth). This finding is particularly salient in the context of the COVID-19 pandemic which resulted in a sense of isolation and loneliness for many persons around the world. Each form of connectedness could be present on its own or several forms of connectedness could present at the same time in each practitioner’s experience. Likewise, the qualities of transcendence, ultimacy, boundlessness, interconnectedness, and positive emotions were cultivated through collective labyrinth walking with a shared intention which expand the work of Pargament et al. (2014).

In a systematic review on labyrinth walking, Davis (2021) reported that four out of seven people experienced positive emotions such as peace when walking a labyrinth individually. The current study expands these findings to the experience of collective labyrinth walking with a shared intention during an international crisis. This collective labyrinth walk cultivated a sense of peace, calm, compassion, relaxation, and increased awareness within a community context. This finding is particularly notable in that the practitioners in our study had been experiencing stress, anxiety, and trauma due to the COVID-19 pandemic and related concerns. The experience of collective labyrinth walking with a shared intention may have served to regulate emotions for the participants in the current study in addition to expanding their awareness.

Although we did not compare people walking with the same intention to those who walk labyrinths with the goal of simply seeing what emerges, we believe that collective labyrinth walking with a shared intention may amplify positive emotions which warrants additional research. This finding provides evidence of the value of labyrinth walking as a social contemplative practice. It suggests that collective labyrinth walking with a shared intention may be a practice that can contribute to emotion regulation during disasters such as the COBID-19 pandemic.

Contemporary contemplative practice may evoke radical shifts in the self and perception of the world, leading to actions that may benefit humanity and the earth. Among the most renowned contemplatives are Thomas Merton, Trappist monk and peace activist; Dorothy Day, social activist and co-founder of the Catholic Worker Movement; Howard Thurman, principal architect of the non-violent civil rights movement and mentor to Martin Luther King, Jr.; Anglican Bishop Desmond Tutu, Anti-Apartheid and human rights activist; and Thich Nhat Hanh, Buddhist monk and peace activist. These are individuals who have taught us that contemplation can foster justice and action (Taylor-Stinson, 2017; Pennington-Russell, 2020). In this study, we verified that this mindset could occur through the practice of collective labyrinth walking with a shared intention.

One branch of The Tree of Contemplative Practices is labeled “activism” and includes practices such as “taking trips to sites of social justice activity, working and volunteering for social causes, attending marches and vigils, and bearing witness” (Kaufman, 2017, p. 5–6). Our findings suggest that collective labyrinth walking with a shared intention can also be a form of contemplative activism where deep insight is cultivated to address the social issues of our time with greater clarity and resolve. Indeed, like other contemplative practices, collective labyrinth walking with a shared intention may serve to cultivate the inner tools for disrupting inner logic system that are based on oppression and bias so that we can see ourselves, others, and the world more clearly (Lee, 2021; Son, 2021). Expanded awareness and vision through this inner work can be transformed into the outer work of supporting social and structural change (Schaarsberg, 2021).

Findings from the current study supported prior work on the use of contemplative practices for social justice concerns (Lee, 2021; Son, 2021). The form of activism present in collective labyrinth walking is more subtle in nature when compared to more well-known or traditional forms of activism such as marching and protesting. Instead, the form of subtle activism we observed in collective labyrinth walking with a shared experience emphasize insight, connection with others and creativity (Hesterman and Hawkey, 2020).

The outer work of social change begins with inner soul work (Lee, 2021). To date, labyrinth walking has primarily focused on inner work (Davis, 2021). The current study expanded this focus to labyrinth walking for communal and cultural flourishing. Further research is needed to explore the ways collective labyrinth walking can be used as a form of contemplative activism.

### Limitations

This study was a community based participatory research (CBPR) study. A CBPR approach views all stakeholders as equal. For this project, stakeholders included the sponsor, an expert practitioner advisory board, the labyrinth community, and the research team. Efforts were made to engage stakeholders at every point in the research process to co-create and shape the project by contributing expertise, sharing in decision making, and taking ownership of the project. This form of research is aimed at increasing knowledge and understanding of a given phenomenon for the purpose of integrating that knowledge in a manner that benefits the community. Inherent in this form of research is the potential for bias as compared to a tightly controlled researcher-initiated project. Although there were participants representing 19 countries, most of these participants were from North America, predominately the United States. Thus, it is not known whether our findings are generalizable cross-culturally. There was also a lack of gender and age diversity with most participants being women in mid-life or older. Therefore, additional research is needed with younger cohorts of practitioners and those who identify as male to with different genders fuller understanding of the impact of practitioners. The data in this project was collected at one time and a longitudinal approach is highly recommended.

### Future research

Future research on collective labyrinth walking with a shared intention could take several directions. For example, there is a need to understand the differences between collective labyrinth walking with a shared intention as compared to individual labyrinth walking without a shared intention. These are both different ways that labyrinth walking can be utilized as a contemplative practice and it is not yet clear how an individual versus a social form of labyrinth walking differs in regards to practitioner experiences and outcomes. Second, comparative research could be conducted between collective labyrinth walking with a shared intention and other well-known practices such as mindful walking with compassion. Although both contemplative practices involve movement (i.e., walking) and compassionate intentions, they are distinct forms of contemplative practice. It is known that labyrinths were constructed based on what has been referred to as sacred geometry. It would be interesting to determine if labyrinth patterns can amplify practitioners’ experiences as compared to other contemplative walking practices which do not hold a similar structure. Quasi-experimental and experimental designs are recommended for further research comparing practices.

Another important area for future directions research is to better understand the mechanisms by which collective labyrinth walking with a shared intention effects individual and social flourishing. For example, the question of possible mediators and moderators by which collective labyrinth walking with a shared intention works is an important step in this line of research. We recommend the use of objective indicators (i.e., psychometric and *in vivo* biomarker measurement) pre- and post- collective labyrinth walking experience to better understand this phenomenon. Ideally, data collection could occur before, during, immediately after, and longitudinally on individual and collective flourishing outcomes.

Finally, research on the effects of walking labyrinths in a collective over time with a shared intention should be examined. For example, what do practitioners report when they participate in collective labyrinth walking with a shared intention over more than one session? Do results change if different intentions are utilized by a collective? Future research should examine not only individual responses but also group responses such as social cohesion between practitioners who are engaging in collective labyrinth walking with a shared intention over time.

## Conclusion

In conclusion, practitioners shared that their experience of collective labyrinth walking with a shared intention allowed them to gain a contemplative posture of peace and calm leading to insights and motivations for positive change during an international crisis. A poignant quote from a participant echoes this sentiment, “The (collective) labyrinth walk reflected the life we are in now…IN this case the wondering about how to come out of this pandemic. The intention made me feel into all the aspects I need to let go of myself to be able to be the intention…of making positive change.” This study highlights the relevance and possibilities of this integrative contemplative practice for cultivating a sense of connection, at multiple levels, and compassion which could potentially bring people together for action that may affect positive change.

## Data availability statement

The datasets presented in this article are not readily available because the data set is confidential per the Institutional Review Board (IRB). Requests to access the datasets should be directed to JM, jocelyn_mcgee@baylor.edu.

## Ethics statement

The studies involving humans were approved by the Baylor University IRB (Reference Number 1711129). The studies were conducted in accordance with the local legislation and institutional requirements. The participants provided their written informed consent to participate in this study.

## Author contributions

JM was the Principal Investigator for the study and oversaw all aspects of the study including contributed to conception and design of the study, organized the database, oversaw statistical analysis, wrote the first draft of the manuscript, and the final version of the manuscript. CK contributed to conception and design of the study, oversaw recruitment efforts, wrote a section of the first draft of the manuscript, and reviewed the final version of the manuscript. SB and RM wrote sections of the manuscript and reviewed the first and final draft of the manuscript. SW reviewed all drafts of the manuscript. All authors contributed to manuscript revision, read, and approved the submitted version.

## Footnotes

1
https://naturesacred.org/sacred_place/american-psychological-association/

2
https://labyrinthsociety.org

3
https://veriditas.org

4
https://aln.org.au
